# Racial Disparities in Hypertension Prevalence within US Gentrifying Neighborhoods

**DOI:** 10.3390/ijerph17217889

**Published:** 2020-10-28

**Authors:** Genee S. Smith, Rachael R. McCleary, Roland J. Thorpe

**Affiliations:** 1 Department of Environmental Health and Engineering, Johns Hopkins Bloomberg School of Public Health, Baltimore, MD 21205, USA; 2 Hopkins Center for Health Disparities Solutions, Johns Hopkins Bloomberg School of Public Health, Baltimore, MD 21205, USA; rmcclear@jhu.edu (R.R.M.); rthorpe@jhu.edu (R.J.T.J.); 3 Program for Research on Men’s Health, Hopkins Center for Health Disparities Solutions, Johns Hopkins Bloomberg School of Public Health, Baltimore, MD 21205, USA; 4 Department of Health, Behavior, and Society, Johns Hopkins Bloomberg School of Public Health, Baltimore, MD 21205, USA

**Keywords:** gentrification, hypertension, health disparities, race, neighborhood

## Abstract

Racial disparities in hypertension remain a persistent public health concern in the US. While several studies report Black–White differences in the health impacts of gentrification, little is known concerning the impact of living in a gentrifying neighborhood on hypertension disparities. Data from the American Community Survey were used to identify gentrifying neighborhoods across the US from 2006 to 2017. Health and demographic data were obtained for non-Hispanic Black and White respondents of the 2014 Medical Expenditure Panel Survey (MEPS) residing in gentrifying neighborhoods. Modified Poisson models were used to determine whether there is a difference in the prevalence of hypertension of individuals by their race/ethnicity for those that live in gentrifying neighborhoods across the US. When compared to Whites living within gentrifying neighborhoods, Blacks living within gentrifying neighborhoods had a similar prevalence of hypertension. The non-existence of Black–White hypertension disparities within US gentrifying neighborhoods underscores the impact of neighborhood environment on race differences in hypertension.

## 1. Introduction

For at least two decades, Blacks have consistently exhibited a higher prevalence of hypertension compared with Whites, roughly 40% and 28%, respectively [1]. While this public health challenge has considerable impact on cardiovascular disease-related morbidity and mortality for Blacks [2], the reasons for these race differences are not fully understood. Implications for work focusing on individual risk factors for hypertension such as diet, physical activity, obesity, etc. have been shown to account for some but not all the observed difference [3], suggesting that other underlying factors, such as the environment in which one lives, contribute to race disparities in hypertension.

A large body of literature exists on the ways neighborhood environment influences risk of hypertension and indicate neighborhood environment as a contributor to racial disparities in hypertension [3,4,5]. Blacks and Whites tend to live in very different neighborhood environments with Blacks more likely to reside in poorer neighborhoods that have less access to services [6]. Studies linking racial residential segregation and neighborhood environment to Black–White hypertension disparities offer concentrated poverty, unequal distribution of resources, and increased stressors as probable mechanisms [7,8,9]. While the effects of segregation and neighborhood environment on racial disparities in hypertension have been acknowledged [3,10,11,12,13], very little is known about the impacts of gentrification on hypertension disparities. Gentrification is the process of working-class neighborhoods being rehabilitated by the middle class, which can result in the displacement of poorer residents [14,15]. In gentrified neighborhoods, Blacks and Whites often experience very different social, political, and financial outcomes [16,17,18]—all factors that contribute to hypertension. Gentrification is often preceded by racial residential segregation; thus, for Blacks the gentrification process can be experienced as a form of structural racism and oppression. This is especially important because in many instances, gentrification does not lead to displacement of native residents but results in gentrifiers and non-gentrifiers residing alongside one another [19,20]. Many of these differences, whether physical, economic, or psychosocial, can result in differential impacts on health, including hypertension [21].

Prior studies on gentrification and health indicate Blacks may fare worse than Whites in these environments. A 2008 Philadelphia survey found that while gentrification was associated with mild improvements in health overall, Blacks residing in gentrified neighborhoods reported poor/fair self-rated health almost twice as much as Whites living in non-gentrified neighborhoods [22]. This study also revealed a difference in health outcomes based on the racial composition of incoming residents associated with gentrification, with “White gentrification” having virtually no effect but “Black gentrification” resulting in slightly worse self-rated health (hinting at longstanding and lingering effects of segregation). Other studies that have explored gentrification and health associations have indicated differential impacts on Blacks and Whites [23,24], reporting increased preterm birth and increased mortality rates only among Blacks [22,25,26]. These findings demonstrate that the health impacts of gentrification can be different among different racial groups. To date, no published studies have examined race disparities in hypertension within US residents living in gentrifying neighborhoods using a national dataset.

The objective of this study was to examine hypertension disparities among Black and White adults who are residing in US gentrifying neighborhoods. We hypothesized that racial hypertension disparities would persist within gentrifying neighborhoods, with higher prevalence of hypertension among Blacks compared to Whites.

## 2. Materials and Methods

### 2.1. Data Source and Sample

We used data from two sources: the 2014 Medical Expenditure Panel (MEPS) Household Component (HC) Survey, and the American Community Survey. The US Census 2006-2010 5-year American Community Survey (ACS 2010 5-year estimate) and the US Census 2013–2017 5-year ACS (2017 5-year estimate) were used to determine whether there is a difference in the prevalence of underlying priority conditions of individuals by their race/ethnicity for those that live in gentrifying neighborhoods across the United States. Based on a sampling frame of the National Health Interview Survey and fielded by the Agency Healthcare Research and Quality, MEPS is a longitudinal survey that collects information on healthcare utilization, expenditures, sources of payment, health insurance coverage, a respondent’s health status, health behaviors, socio-economic demographic characteristics, and employment status on noninstitutionalized civilian US population [27]. MEPS consists of five interviews conducted over a 2½-year period and is unique in its ability to link data on individuals and households (including demographics, health status, employment, and income) to information on their use of health services. Additional details are described elsewhere [27].

The American Community Survey (ACS), conducted by the US Census Bureau, collects relevant data about the United States population and housing characteristics. Annual information collected includes demographic, social, economic, and housing regarding communities across the US and Puerto Rico. A key feature of the ACS is that it uses monthly samples to produce yearly updated estimates for small areas such as census tracts and census block groups. These estimates are produced as 1-, 3- or 5-year estimates. Details of the ACS can be found elsewhere [28]. We extracted both the ACS 2010 5-year and the ACS 2017 5-year estimate data as two data points in order to measure the change of a neighborhood over time. For simplicity, we refer to data from the ACS 2010 5-year as the “start point” and data from the ACS 2017 5-year as the “end point”.

For our analysis, we identified 887 White and Black respondents from MEPS who were 18 years of age or older and who lived in neighborhoods that were considered gentrifying. Deriving methods from Ding et al. [29], a respondent was considered to have lived in a “gentrifying neighborhood” if (1) the median household income of the Census Tract they reside in at the start point was less than the median household income of the corresponding Core Based Statistical Area (CBSA) at the start point; (2) the median gross rent of the Census Tract at the end point was greater than the median gross rent of the CBSA at the end point, or the median household income of the Census Tract at the end point was greater than the median household income of the CBSA at the end point; and (3) the percentage of college-educated (obtained at least a bachelor’s degree) residents in the Census Tract at the end point was greater than the percentage of college-educated residents in the CBSA at the end point.

### 2.2. Outcomes

The outcome measure for this study was hypertension. Participants reported whether they were ever told by a healthcare provider if they had been diagnosed with high blood pressure (excluding during pregnancy). A binary variable was used to identify those individuals who were diagnosed with hypertension.

### 2.3. Independent Variables

The main independent variable for this study was race and ethnicity. Participants reported their race as White, Black, American Indian/Alaskan Native, Asian, Native Hawaiian/Pacific Islander or Multiple races. Participants reported their ethnicity as Hispanic or Not Hispanic. For this study, we identified individuals who reported their ethnicity as Non-Hispanic and their race as White or Black.

### 2.4. Covariates

Covariates—demographic variables and health-related characteristics—in this study were based on prior literature [2,4,5,12]. Demographic variables included age, gender, education, poverty status, and employment status. Ages of the participants were categorized into three groups: 18 to 39, 40 to 59, and 60 or over. Female gender was coded as a dichotomous variable. Participants reported their education level as having obtained an education of grade 8 or below, some high school, high school diploma or GED, some college, bachelor’s degree, or an advanced degree at study inception. Poverty status was derived from MEPS by dividing the family income by the appropriate poverty line and then classifying each participant into one of five categories [27]. These categories are (1) poor/negative (less than 100%), (2) near poor (100% to less than 125%), (3) low income (125% to less than 200%), (4) middle income (200% to less than 400%), and (5) high income (greater than or equal to 400%). Employment status was determined on the participant report of whether they indicated if they were employed at the start time of the interview.

Health-related characteristics included health insurance status, smoking status, physical activity level, weight classification, self-rated health, and diabetes. The MEPS respondent’s health insurance status was indicated by having either private insurance coverage, public insurance coverage, or uninsured status. The respondent’s smoking status was determined by whether or not they indicated that they currently smoke. Physical activity level was based on whether or not they currently spend a half hour or more in moderate to vigorous physical activity at least 5 times a week. The participants were classified as being underweight (body mass index, BMI less than 18.5), normal weight (BMI 18.5 to less than 25.0), overweight (BMI 25.0 to less than 30.0), or obese (BMI 30.0 or higher) according to Centers for Disease Control and Prevention classification. Favorable self-rated health was defined as the MEPS respondent indicating that their perceived health status was excellent, very good, or good, with unfavorable self-rated health status defined as the respondent indicating that their perceived health status was fair or poor. Diabetes status was based on each MEPS respondent’s self-report of whether or not they received a diagnosis of diabetes.

### 2.5. Analysis

The mean and proportional differences between racial categories for the demographic and health-related characteristics were evaluated using Student’s *t* for continuous variables and chi-square tests for categorical variables, respectively. Because the prevalence of hypertension is greater than 10%, modified Poisson models, rather than logistic regression models, were used to examine the relation between hypertension and Non-Hispanic White and Black respondents who resided in gentrifying neighborhoods [30,31,32]. Three models were used to estimate the association between race and hypertension. The first model included biological factors (age and gender). The second model included all variables in Model 1 along with socioeconomic factors (education, income category, employment, and insurance status). The third model included all variables in Model 2 and health factors (smoking status, physical activity, weight status, diabetes, and self-rated health). Survey weights and design factors were invoked to account for the complex sampling design of MEPS. Stata 15.0 SE was used to analyze these data. *p*-values less than 0.05 were considered statistically significant.

## 3. Results

In Table 1, we report demographic and health characteristics of MEPS participants living in gentrifying neighborhoods by race. Blacks living in gentrifying neighborhoods were more likely to be uninsured (12.8% vs. 5.4%; *p* = 0.006) and obese (35.6% vs. 23.8%; *p* = 0.005) than their White counterparts. Age, gender, education level, employment status, and health characteristics (smoking status, physical activity, self-rated health, and diabetes) were similar among Black and White study participants.

The association between race and hypertension among MEPS adult participants residing in gentrifying neighborhoods is presented in Table 2. In model 1, Blacks living within gentrifying neighborhoods had a 26% higher prevalence of hypertension (95% confidence interval (CI): 1.02–1.56) as compared to Whites adjusting for age and gender. After further adjusting for education, income category, employment status, and insurance status in Model 2, Blacks living within gentrifying neighborhoods still had a 26% higher prevalence (95% CI: 1.03–1.54) of hypertension relative to Whites living in gentrifying neighborhoods. In the fully adjusted model, which included health characteristics such as smoking status, physical activity, weight status, diabetes, and self-rated health, Blacks living in gentrifying neighborhoods had a similar prevalence of hypertension (PR: 1.15; 95% CI: 0.94, 1.41) compared with Whites living in gentrifying neighborhoods.

## 4. Discussion

Racial/ethnic differences in hypertension prevalence within the US have been well-documented. However, the impact of neighborhood environment on racial hypertension disparities has been studied to a lesser extent. Using data from the 2014 MEPS, we examined Black–White differences in hypertension among residents living within gentrifying neighborhoods. Overall, when compared to Whites living within gentrifying neighborhoods, Blacks living within gentrifying neighborhoods had a similar prevalence of hypertension. These findings highlight the importance of gentrification in racial/ethnic hypertension disparities and indicate a need for further research identifying drivers of these associations to inform public policy.

A growing body of research suggests an association between neighborhood environment and racial hypertension disparities. A study of neighborhood context and hypertension disparities by Morenoff et al. [4] found a negative association between hypertension and neighborhood affluence/gentrification in Chicago; however, the gentrification definition (i.e., areas with high proportion of residential turnover, young adult population, educated residents, and professional or managerial employment) used was conflated with affluence. Gentrification is not necessarily akin to affluence but often spurred by artists and students no better off financially than existing residents and commonly results in neighborhood transformations to middle class standards [33]. Still, Morenoff et al. identified highly significant Black–White hypertension disparities that were eliminated following adjustment for neighborhood context. The EHDIC (Exploring Health Disparities in Integrated Communities) study in Baltimore found that racial disparities in hypertension prevalence were smaller for residents living in similar despondent neighborhood conditions [12]. While our study population lived in neighborhoods with an above-the-median household income, both studies showed that when Blacks and Whites live in similar conditions, racial hypertension disparities dissolve [5]. Our findings are also consistent with an analysis of National Health and Nutrition Examination Survey data which revealed smaller racial hypertension disparities in neighborhoods where Blacks and Whites lived together than in racially segregated neighborhoods [7]. However, as other studies examining the impacts of “Black gentrification” and “White gentrification” have indicated, it is possible that the racial hypertension disparities could vary based upon the race/ethnicity of the gentrifying population. Prior literature suggests that racial health disparities persist within gentrified neighborhoods [23,24,25,26], but our findings indicated the expansion or elimination of health disparities within gentrifying neighborhoods differs across health outcomes.

Much of the variation in Black–White hypertension prevalence in the US can be explained by the conditions in which people live, such as racial residential segregation, stressors, community social deprivation, etc. [7,9,34]. Hypertension is lower in neighborhoods that have increased safety, walkability, social cohesion, and access to healthy foods [3]. Thus, one plausible explanation of why hypertension disparities no longer persist within gentrifying neighborhoods is that these neighborhoods have characteristics that promote healthier behaviors, reducing Black–White disparities in hypertension. Gentrifying neighborhoods may also have more equitable access to neighborhood resources such as gyms and healthcare facilities, which could aid in reducing health disparities. Gentrifying neighborhoods are also frequently accompanied by recent changes such as new amenities, investments and resources, residential improvements, and increased political power. It is possible that these attributes act as a barrier to stress—a risk factor for hypertension. This notion is supported by results from a multilevel analysis conducted by Gibbons in Pennsylvania which found that among residents living within gentrifying neighborhoods, Blacks were overall less likely than Whites to report high levels of self-rated stress [35].

We acknowledge limitations within this study. This investigation utilized a cross-sectional study design, measuring all variables during a snapshot in time; thus, our findings do not infer causality. While unable to determine whether the elimination of Black–White hypertension remains over time, we recognize that gentrification is a process and not a static condition. Future studies using longitudinal designs that can examine gentrification at different time points would strengthen causal inference. Our static assessment of the race–hypertension association within gentrifying neighborhoods also prevented distinguishing new from existing residents and examining the impacts of the racial composition of gentrifiers on hypertension prevalence (i.e., “Black gentrification” vs. “White gentrification”). For this study, we also defined gentrification using census tracts, including those outside of primary or secondary cities of CBSAs. With methods for gentrification measurement still debated in the literature, each option will inherently include drawbacks and can result in drawing different conclusions [36,37,38]. While our use of census tracts does provide an easily accessible, repeatable, and validated method for defining neighborhoods, these boundaries are not always consistent with the lived experience of residents. Given variation across metropolitan areas, this approach to defining gentrifying neighborhoods likely includes new-build and rural gentrification [38]. Additionally, we were unable to assess factors that may point toward the mechanism through which racial hypertension disparities are eliminated in gentrifying neighborhoods. Understanding how neighborhood characteristics (such as greenspace, walkability, healthcare access, social networks, etc.) impact these findings can inform public policy [39]. Studies incorporating various measures from the built, natural, and social environment are warranted. Finally, our outcome of interest is self-reported diagnosed hypertension. Racial disparities may persist among undiagnosed hypertensive residents in gentrifying neighborhoods.

In our final, fully adjusted model, we found no association between race and hypertension among Black and White adults living within US gentrifying neighborhoods. This is consistent with previous research examining neighborhood context and hypertension [4,12]. It is worth noting that when accounting for biologic and socioeconomic factors only, Blacks living within gentrifying neighborhoods had a higher prevalence of hypertension relative to Whites living in gentrifying neighborhoods. However, when we included the health factors (smoking status, physical activity, weight status, diabetes, and self-rated health), the association between race and hypertension went away. Future research seeking to explain these race differences in hypertension prevalence will make a significant contribution to this area of research.

This investigation is an important contribution to the growing body of literature on neighborhood environment and racial disparities in hypertension. To our knowledge, this is the first nationwide study to examine Black–White disparities in hypertension among residents of US gentrifying neighborhoods. Our use of MEPS yields individual-level data on a nationally representative sample of non-institutionalized US adults. Additionally, the use of MEPS data allows for linkage to link to ACS data at the census block level and provides health information for sufficient numbers of Black and Whites residing in US gentrifying neighborhoods.

## 5. Conclusions

We found that Black–White disparities in hypertension prevalence were non-existent among US residents living within gentrifying neighborhoods. These findings point toward the environment in which people live, as opposed to race itself, as a seminal reason for the persistence of racial hypertension disparities. As gentrification continues throughout the US, more research will be needed to understand the mechanisms through which these disparities dissipate, and what if any gentrifying conditions may exacerbate hypertension prevalence, awareness, treatment, and control. A deeper understanding of the relationship between neighborhood gentrification and hypertension can inform both targeted hypertension interventions and public policies on equitable neighborhood redevelopment, ultimately reducing racial hypertension disparities.

## Figures and Tables

**Table 1:** Demographic and health characteristics of Medical Expenditure Panel Survey respondents living within gentrifying neighborhoods by race, 2014.

Characteristics		Non-Hispanic Whites	Non-Hispanic Blacks	*p*-value
		(*n* = 689)	(*n* = 247)	
Age (%)				0.352
	18–39 years	45.0	50.5	
	40–59 years	27.8	28.9	
	60+ years	27.2	20.6	
Female (%)		48.0	51.2	0.447
Education (%)				0.677
	Grade 8 or below	1.6	2.3	
	Some High School	8.8	8.5	
	High School Diploma or GED	17.2	22.7	
	Some College	32.5	31.5	
	Bachelor’s Degree	25.0	22.2	
	Advanced Degree	14.8	13.0	
Income Category (%)				0.644
	Poor/Negative	9.9	13.1	
	Near Poor	5.8	3.9	
	Low Income	11.7	9.0	
	Middle Income	26.5	28.6	
	High Income	46.2	45.3	
Employed (%)		67.5	74.6	0.149
Insurance Status (%)				0.007
	Private	76.7	63.8	
	Public	17.9	23.4	
	Uninsured	5.4	12.8	
Weight Status				0.005
	Underweight	2.7	0.4	
	Normal	39.6	28.7	
	Overweight	33.9	35.3	
	Obese	23.8	35.6	
Current Smoker (%)		13.8	15.2	0.655
Physically Active (%)		59.3	52.5	0.155
Good/Excellent Self-Rated Health (%)		90.5	88.7	0.535
Diabetes (%)		7.4	8.6	0.625

**Table 2:** Prevalence ratios and 95% confidence intervals of hypertension among Medical Expenditure Panel Survey respondents, age 18 years and older, living within gentrifying neighborhoods, 2014.

Characteristics	Model 1	Model 2	Model 3
Black	1.26 (1.02, 1.56)	1.26 (1.03, 1.54)	1.16 (0.95, 1.42)
Age			
18–39 years	1.00	1.00	1.00
40–59 years	3.09 (1.89, 5.07)	3.04 (1.85, 5.01)	2.47 (1.51, 4.04)
60+ years	5.52 (3.72, 8.20)	4.62 (3.07, 6.97)	3.40 (2.21, 5.24)
Female	0.82 (0.66, 1.01)	0.79 (0.64, 0.97)	0.74 (0.59, 0.94)
Education			
Grade 8 or below		1.04 (0.73, 1.49)	1.06 (0.70, 1.59)
Some High School		0.98 (0.70, 1.35)	0.99 (0.69, 1.42)
High School Diploma or GED		1.00	1.00
Some College		0.95 (0.71, 1.25)	0.92 (0.68, 1.24)
Bachelor’s Degree		0.91 (0.64, 1.30)	0.93 (0.62, 1.37)
Advanced Degree		0.71 (0.47, 1.07)	0.73 (0.46, 1.14)
Income Category			
Poor/Negative		1.00	1.00
Near Poor		1.29 (0.80, 2.07)	1.21 (0.75, 1.97)
Low Income		1.32 (0.78, 2.21)	1.19 (0.74, 1.93)
Middle Income		1.24 (0.77, 1.98)	1.29 (0.81, 2.04)
High Income		1.34 (0.81, 2.21)	1.42 (0.85, 2.37)
Employed		0.75 (0.61, 0.94)	0.71 (0.55, 0.92)
Insurance Status			
Private		1.00	1.00
Public		1.22 (1.00, 1.48)	1.15 (0.94, 1.40)
Uninsured		1.03 (0.71, 1.49)	0.94 (0.65, 1.36)
Current Smoker			0.82 (0.58, 1.16)
Physically Active			0.92 (0.73, 1.16)
Weight Status			
Underweight/Normal			1.00
Overweight			1.11 (0.77, 1.60)
Obese			1.55 (1.11, 2.17)
Diabetes			1.43 (1.13, 1.80)
Good/Excellent Self-Rated Health			0.95 (0.72, 1.26)

## References

[1] Fryar C., Ostchega Y., Hales C.M., Zhang G., Kruszon-Moran D. (2017). Hypertension Prevalence and Control Among Adults: United States, 2015–2016.

[2] Hertz R.P., Unger A.N., Cornell J.A., Saunders E. (2005). Racial disparities in hypertension prevalence, awareness, and management. Arch. Intern. Med..

[3] Mujahid M.S., Roux A.V.D., Morenoff J.D., Raghunathan T.E., Cooper R.S., Ni H., Sheaf S. (2008). Neighborhood characteristics and hypertension. Epidemiology.

[4] Morenoff J.D., House J.S., Hansen B.B., Williams D.R., Kaplan G.A., Hunte H.E. (2007). Understanding social disparities in hypertension prevalence, awareness, treatment, and control: The role of neighborhood context. Soc. Sci. Med..

[5] LaVeist T., Pollack K., Thorpe R., Fesahazion R., Gaskin D. (2011). Place, not race: Disparities dissipate in southwest Baltimore when blacks and whites live under similar conditions. Health Aff..

[6] LaVeist T.A., Gaskin D.J., Richard P. (2009). The economic burden of health inequalities in the United States. Int. J. Health Serv..

[7] Kershaw K.N., Diez Roux A.V., Burgard S.A., Lisabeth L.D., Mujahid M.S., Schulz A.J. (2011). Metropolitan-level racial residential segregation and black-white disparities in hypertension. Am. J. Epidemiol..

[8] Jones A. (2013). Segregation and cardiovascular illness: The role of individual and metropolitan socioeconomic status. Health Place.

[9] Mujahid M.S., Roux A.V.D., Cooper R.C., Shea S., Williams D.R. (2011). Neighborhood stressors and race/ethnic differences in hypertension prevalence (the Multi-Ethnic Study of Atherosclerosis). Am. J. Hypertens..

[10] Kershaw K.N., Robinson W.R., Gordon-Larsen P., Hicken M.T., Goff D.C., Carnethon M.R., Kiefe C.I., Sidney S., Roux A.V.D. (2017). Association of changes in neighborhood-level racial residential segregation with changes in blood pressure among black adults: The CARDIA study. JAMA Intern. Med..

[11] LaVeist T.A. (2005). Disentangling race and socioeconomic status: A key to understanding health inequalities. J. Urban Health.

[12] Thorpe R.J., Brandon D.T., LaVeist T.A. (2008). Social context as an explanation for race disparities in hypertension: Findings from the Exploring Health Disparities in Integrated Communities (EHDIC) Study. Soc. Sci. Med..

[13] Usher T., Gaskin D.J., Bower K., Rohde C., Thorpe R.J. (2018). Residential segregation and hypertension prevalence in black and white older adults. J. Appl. Gerontol..

[14] Smith N. (1982). Gentrification and uneven development. Econ. Geogr..

[15] Lees L., Slater T., Wyly E. (2013). Gentrification.

[16] Mehdipanah R., Marra G., Melis G., Gelormino E. (2017). Urban renewal, gentrification and health equity: A realist perspective. Eur. J. Public Health.

[17] Slater T. (2006). The Eviction of Critical Perspectives from Gentrification Research. Int. J. Urban Reg. Res..

[18] Hwang J. (2015). Gentrification in changing cities: Immigration, new diversity, and racial inequality in neighborhood renewal. Ann. Am. Acad. Political Soc. Sci..

[19] Freeman L. (2011). There Goes the Hood: Views of Gentrification from the Ground Up.

[20] Hyra D.S. (2017). Race, Class, and Politics in the Cappuccino City.

[21] Bell C.N., Thorpe R.J., Bowie J.V., LaVeist T.A. (2018). Race disparities in cardiovascular disease risk factors within socioeconomic status strata. Ann. Epidemiol..

[22] Gibbons J., Barton M.S. (2016). The association of minority self-rated health with black versus white gentrification. J. Urban Health.

[23] Smith G.S., Breakstone H., Dean L.T., Thorpe R.J. (2020). Impacts of gentrification on health in the united states: A Systematic review of the literature. J. Urban Health.

[24] Izenberg J.M., Mujahid M.S., Yen I.H. (2018). Health in changing neighborhoods: A study of the relationship between gentrification and self-rated health in the state of California. Health Place.

[25] Huynh M., Maroko A.R. (2014). Gentrification and preterm birth in New York City, 2008–2010. J. Urban Health.

[26] Just Cause (2015). Development without Displacement: Resisting Gentrification in the Bay Area.

[27] Agency for Healthcare Research and Quality (2019). Medical Expenditure Panel Survey.

[28] United States Census Bureau (2020). American Community Survey (ACS). https://www.census.gov/programs-surveys/acs.

[29] Ding L., Hwang J., Divringi E. (2016). Gentrification and residential mobility in Philadelphia. Reg. Sci. Urban Econ..

[30] Zou G. (2004). A modified poisson regression approach to prospective studies with binary data. Am. J. Epidemiol..

[31] McNutt L.-A., Wu C., Xue X., Hafner J.P. (2003). Estimating the relative risk in cohort studies and clinical trials of common outcomes. Am. J. Epidemiol..

[32] Thorpe R.J., Parker L.J., Cobb R.J., Dillard F., Bowie J. (2017). Association between discrimination and obesity in African-American men. Biodemogr. Soc. Biol..

[33] Chapple K., Zuk M. (2016). Forewarned: The use of neighborhood early warning systems for gentrification and displacement. Cityscape.

[34] Claudel S.E., Adu-Brimpong J., Banks A., Ayers C., Albert M.A., Das S.R., de Lemos J.A., Leonard T., Neeland I.J., Rivers J.P. (2018). Association between neighborhood-level socioeconomic deprivation and incident hypertension: A longitudinal analysis of data from the Dallas heart study. Am. Heart J..

[35] Gibbons J. (2019). Are gentrifying neighborhoods more stressful? A multilevel analysis of self-rated stress. SSM Popul. Health.

[36] Firth C.L., Fuller D., Wasfi R., Kestens Y., Winters M. (2020). Causally speaking: Challenges in measuring gentrification for population health research in the United States and Canada. Health Place.

[37] Mujahid M.S., Sohn E.K., Izenberg J.M., Gao X., Tulier M.E., Lee M.M., Yen I.H. (2019). Gentrification and displacement in the san francisco bay area: a comparison of measurement approaches. Int. J. Environ. Res. Public Health.

[38] Liu C., Deng Y., Song W., Wu Q., Gong J. (2019). A comparison of the approaches for gentrification identification. Cities.

[39] Smith G.S., Thorpe R.J. (2020). Gentrification: A Priority for environmental justice and health equity research. Ethn. Dis..

